# Community Drivers Affecting Adherence to WHO Guidelines Against COVID-19 Amongst Rural Ugandan Market Vendors

**DOI:** 10.3389/fpubh.2020.00340

**Published:** 2020-07-03

**Authors:** Ibe Michael Usman, Fred Ssempijja, Robinson Ssebuufu, Ann Monima Lemuel, Victor Bassey Archibong, Emmanuel Tiyo Ayikobua, Joshua Ojodale Aruwa, Stellamaris Kembabazi, Eric Simidi Kegoye, John Tabakwot Ayuba, Olatayo Segun Okeniran, Isaac Echoru, Azeez Adeoye, Regan Mujinya, Viola Nankya, Keneth Iceland Kasozi

**Affiliations:** ^1^Faculty of Biomedicals Sciences, Kampala International University Western Campus, Bushenyi, Uganda; ^2^Faculty of Clinical Medicine and Dentistry, Kampala International University Teaching Hospital, Bushenyi, Uganda; ^3^Department of Physiology, School of Health Sciences, Soroti University, Soroti, Uganda; ^4^School of Medicine, Kabale University, Kabale, Uganda; ^5^School of Nursing, Kampala International University Teaching Hospital, Bushenyi, Uganda; ^6^Infection Medicine, Deanery of Biomedical Sciences, College of Medicine and Veterinary Medicine, The University of Edinburgh, Edinburgh, United Kingdom

**Keywords:** COVID-19, SARS CoV-2, market-vendors, information on COVID-19, rural community, Africa response to COVID-19, COVID-19 in Uganda

## Abstract

**Background:** Market vendors occupy a strategic position in the fight against the spread of SARS CoV-2 in rural Uganda. To successfully contain the spread of the virus, special attention needs to be given to this set of people by assessing the type of information, source of information, and practices they inculcate as regards adherence to WHO guidelines in the fight against COVID-19 in Uganda. The study aimed to assess the role of information sources, education level, and phone internet connectivity in influencing COVID-19 knowledge among the rural market vendors; and the relationship existing between knowledge, attitude, and practices among them.

**Methods:** The study was a descriptive cross-sectional study among rural market vendors (*n* = 248) in southwestern Uganda. Information was collected using a questionnaire and descriptively presented as frequency and percentages.

**Results:** The study showed that the majority of the rural market vendors had sufficient information regarding COVID-19 with the majority being female individuals and have attained a secondary level of education, The general percentage score for knowledge, attitude, and practices were (75.57, 82.6, and 76.50% respectively). There was a positive correlation between attitude and practices (*r* = 0.17, *p* = 0.007), as well as their knowledge with practices (*r* = 0.29, *p* < 0.001). The majority of the people in the population did not have their phones connected to the internet (OR = 1.96, 95%CI: 1.16–3.31, *P* = 0.01). The majority of people received their information regarding COVID-19 from one source (radio) (OR = 1.55).

**Conclusion:** Where and how the rural market vendors get their information and education level are vital in breaking COVID 19 infection circle in line with WHO guidelines. Therefore, sources of information and education level played a key role in molding their knowledge and practices. However, the level of knowledge on COVID 19 among our respondents was not linked with phone internet connectivity.

## Introduction

The COVID-19 outbreak in Wuhan was traced to seafood markets in Wuhan in December 2019 suggesting that the virus jumped from sea animals to humans (1). The outbreak of COVID-19 triggered a global response by all countries of the world in general and the East African Community (EAC) in particular to fight the common enemy (2). With SARS CoV-2 spreading at an alarming rate than can be controlled globally, Uganda and other EAC are doing their best to contain the pandemic by following guidelines published by the World Health Organization (WHO) (3).

Market vendors in all East African countries have been identified as crucial workers whose activities provide lifeline support during the COVID-19 lockdown across the region, through access to essential commodities like food, mobile money, etc. (4). In Uganda, market vendors continue in business while their colleagues in other sectors are home during this COVID-19 lockdown period (4). The services provided by this informal sector eases access to food and money that are very vital for human survival especially during the lockdown (5). The continued activities of the market vendors during the lockdown places them at a greater risk because they continue to interact with many people from the general public (6). The Uganda government established regulations in line with the WHO and Ugandan Ministry of Health guidelines, to protect market vendors and the general public from the virus. However, the architectural design of rural markets in Uganda makes it difficult to implement some of these guidelines, such as regular washing of hands, social distancing, use of hand sanitizer (7). Special attention is needed to regulate and monitor market vendors to ensure they follow guidelines to prevent the spread of outbreaks (8) such as COVID-19. Market vendors across EAC have a similar pattern of activities and are considered to be generally less knowledgeable and careless about public health problems (9). An explosion of COVID-19 cases among the market vendors can set EAC member states on edge yet most of the studies on COVID-19 do not capture them (10). Hence the need to identify factors that could influence the adequate implementation of the Ugandan Ministry of Health and WHO guidelines (11, 12) among rural market vendors in Uganda.

To overcome the challenges posed by the inability to comply with the control measures of pandemics in the open markets of Uganda, it was essential to identify socio-economic drivers affecting practice and effective implementation of the WHO guidelines among vendors (12). Women constitute a significant proportion of vendors in most African rural markets because women are more inclined to vending since many are single mothers with mouths to feed (13, 14). Women and girls have been identified as vulnerable people due to their low decision-making authority in rural communities of Africa and this makes them important in community disease response projects due to their added responsibilities caring for children and the elderly in society, and in caring for the sick in at home (15). A possible sex difference in vulnerability to COVID-19 needs to be given keen attention. An ongoing survey in urban slums of Kenya suggested that gender differences need to be looked into with regards to COVID-19 (16). A recent study confirmed the possible gender difference in curbing epidemics in informal settlements (15).

Knowledge is crucial in shaping people's behavior and practices especially during any disease outbreak (17). This is because knowledge level is linked with panic emotion among most populations, which in turn influences their attitude and practices toward COVID-19 (18). The possible factors that could influence the knowledge, attitude, and practices include but not limited to sex, sources of information, and education level (17, 19). Knowledge of the market vendors regarding COVID-19 and how they get information is a key measure in the fight against SARS CoV-2, however, a scarcity of epidemiological studies from rural communities in Africa created a rationale for this study. This was timely due to the threat of misinformation and fake news (20). Misinformation among market vendors can be more disastrous and may act as a tool to worsen the epidemiologic characteristics of the pandemic since a rise in COVID 19 cases among them will expose the local community to an increased risk of infection (21, 22). Given the present challenge, the major sources of information regarding the COVID-19 pandemic could either guarantee success or compromise the fight, especially among market vendors who are mostly rural settlers (23). Possible sources of information available to the market vendors across Uganda include; friends, radios, televisions, social media, newspapers (22), and the source (s) of information used is vital in determining effectiveness in receiving and interpreting COVID-19 knowledge among them (17).

The internet is often not mentioned as a source of information among market vendors, possibly as a result of the high cost of phones with internet connectivity, but notwithstanding its importance in the dissemination of vital information cannot be dispelled (24). Phone internet connectivity has been deployed in different places around the globe to curb the spread, diagnosis, and management of COVID-19 (25–27) due to improved awareness about COVID-19 coming with regular updates from different internet applications. The National health authorities and the WHO have been taking advantage of the internet in fighting disinformation on COVID-19 through counternarratives online and offline (28). The Ugandan Ministry of health at a specific time has used social media to dispel unnecessary panics, associated with false reports of COVID-19 cases (28). In essence, internet connectivity could serve as a portal for disseminating reliable information on the pandemic as well as enable citizens to report suspected cases. Internet tools such as social media should be incorporated in the prevention of COVID-19 because the majority of the vendors are youth across the eastern African region (26).

Education level and professional training are very instrumental in shaping people's knowledge, attitudes, and practices regarding COVID-19 (29). In creating preventive and mitigation measures for COVID-19, attention needs to be given to the educational level of the market vendors (15). A Chinese study showed that education level, professional training, and relevant COVID-19 training were very instrumental in shaping peoples knowledge, attitude and practices regarding COVID19 (29), and this is in line with the global norm where good knowledge, attitude, and practices were associated with qualifications of individuals among the different continents (15). Educational level is an indicator of poverty as a risk factor, which in itself is a risk factor to the spread of every form of infection (15). Therefore, the knowledge levels of market vendors toward COVID-19 need to be assessed to control SARS CoV-2 by looking at the different dynamics of the disease such as the source of information, internet, education levels and how these are related to their knowledge on the mode of spread, prevention, symptoms and signs, and possible management strategies. Their knowledge regarding these aspects directly or indirectly molds their attitude and practices especially during this pandemic (30).

The study aimed to assess the role of information sources, education level, and phone internet connectivity in influencing COVID-19 knowledge among the rural market vendors; and the relationship existing between knowledge, attitude, and practices among them.

## Methods

### Study Site and Design

The study design was a descriptive cross-sectional study among market vendors in the Ishaka-Bushenyi municipality of south-western Uganda. A Simple random sampling technique was employed.

### Study Population

#### Inclusion Criteria

The study population is market vendors in the Ishaka-Bushenyi municipality in south-western Uganda. They include sellers of fruits, vegetables, and food store owners who were allowed to continue their businesses during the lockdown order by the Ugandan government; this was because they were considered as sellers of essential commodities.

#### Exclusion Criteria

People in the market not who are not vending during the nationwide lockdown and those who refused to give their consent for the studies were excluded from the present studies.

### Data Collection and Measures

A closed-ended pretested questionnaire comprising multiple-choice questions was employed. The questionnaire measured sociodemographic data (age, sex, educational level, and religion), knowledge, attitude, and practices. The questionnaire was reviewed and validated by different experts. The questionnaire was uploaded on the google form (via docs.google.com/forms), and the link shared among assessors to minimize paper printing and social distancing, also masks were worn as an extra protective measure. Introductory letters addressed to the market authorities from Local Council were given to the assessors detailing the purpose of the study. Each of the assessors was assigned a Ruyankole interpreter. A total of 248 rural market vendors were recruited for the study.

### Study Variables

#### Independent Variables

Demographic details which include age, gender, educational level, marital status, religion. Internet connectivity, sources of information on COVID-19 and educational status.

#### Dependent Variables

Knowledge, attitude, and practices toward COVID-19.

##### Knowledge

Our study assessed knowledge on specific facts regarding COVID-19 developed in line with WHO guidelines (31) and modified to suit market vendors. These questions include the following; there is no effective cure for COVID-19 at the moment, early identification of symptoms and supportive care can help most patients recover from the infection, not all persons with COVID-19 will develop to severe cases, elderly people, people with underlying chronic illnesses and the obese are more likely to develop severe cases of the infection, eating or contacting wild animals would result in the infection by the SARS CoV-2, persons with COVID-2019 can infect others when a fever is not present, SARS CoV-2spreads via respiratory droplets of infected individuals, it is necessary for children and young adults to take measures to prevent the infection by the SARS CoV-2), isolation and treatment of people who are infected with the SARS CoV-2 are effective ways to reduce the spread of the virus, people with previous contact with someone infected with the SARS CoV-2should be immediately isolated in a proper place for 14 days' observation. Each correct response weighed 1 point and 0 for incorrect responses. The higher the points, the more knowledge the market vendors are had on COVID 19.

##### Attitude

The attitude among rural market vendors was assessed using 5 questions that have been adopted from (32) and modified appropriately for COVID-19 by the authors. The responses were; Yes and No. Some questions were reversed to eliminate biases of giving a single similar response in all the items. Response showing positive attitude were assigned 1 and negative attitude were assigned 0.

##### Practices

Practices were assessed using seven questions. These questions were developed based on the WHO and Ministry of Health Uganda recommendation for practices on prevention of COVID-19 transmission i.e., hand washing, use of hand sanitizers, avoiding crowded places, maintenance of social distance, cleaning of surfaces with soap and bleach, use of face mask, covering of your mouth with handkerchief, elbow or tissue paper when sneezing (31). The responses were; Yes and No. Response showing good practices were assigned 1 and bad practices were assigned 0.

### Data Management and Analysis

Fully completed questionnaires were extracted from Google Forms and exported to a Microsoft Excel 2016 for cleaning and coding. The cleaned data was exported to IBM SPSS Statistics 20 and GraphPad 8.3 for analyses. Categorical data were summarized as frequencies and proportions. Associations between independent variables and dependent variables were assessed using multivariate analysis in Winpepi software. One-Way Analysis of Variance and Tukey *post-hoc* test; were done using GraphPad Prism 8.3 to compare KAPs against the independent variables. The sum score of each outcome was assessed based on Bloom's cut off point (33). Based on the sum scores, level of knowledge was classified into low-level knowledge (<60%; 0–8 scores), moderate level knowledge (60–80%; 9–11 scores), and high-level knowledge (80–100%; 12–15 scores). The scores for attitude were classified into positive attitude (80–100%; 60–75 scores), neutral attitude (60–80%; 45–59 scores), and negative attitude (<60%; 15–44 scores). The level of practice was classified into poor-level (<60%; 10–29 scores), fair level (60–80%; 30–40 scores), and good level (80–100%; 41–50 scores). Spearman correlation was used to assess the relationship between knowledge, attitude, and practices using IBM SPSS Statistics 20. Values were considered statistically significant at *p* < 0.05.

## Results

### Sociodemographic Characteristics of the Study Population

The Majority of our respondents are female 154/248 (62.1%) and falls within 21–30 age categories 131/248 (52.8). The majority of our respondents had attained secondary level 134/248 (54.0%) and most of our respondents were single 123/248 (49.6%). Protestants accounted for 99/248 (39.9%) of our respondents (Table 1).

**Table 1:** Sociodemographic characteristics of the study population.

		**Frequency (%)**	**95% CI**
Age categories in years	<21	33 (13.3)	9.5–18.0
	21–30	131 (52.8)	46.6–58.2
	31–40	60 (24.2)	19.2–29.8
	41–50	16 (6.5)	3.9–10.1
	51–60	3 (1.2)	0.3–3.3
	>60	2 (0.8)	0.1–2.6
	Undeclared	3 (1.2)	0.3–3.3
Gender	Male	94 (37.9)	32.0–44.1
	Female	154 (62.1)	56.0–68.0
Educational status	No formal education	5 (2.0)	0.7–4.4
	Primary level	73 (29.4)	24.0–35.0
	Secondary level	134 (54.0)	47.8–60.2
	Tertiary level	36 (14.5)	10.5–19.3
Marital status	Divorced	2 (0.8)	0.1–2.6
	Married	122 (49.2)	43.0–55.4
	Single	123 (49.6)	43.4–55.8
	Widow	1 (0.4)	0.0–2.0
Religion	Adventists	13 (5.2)	3.0–8.6
	Catholics	85 (34.3)	28.6–40.4
	Muslims	29 (11.7)	8.1–16.2
	Protestant	99 (39.9)	34.0–46.1
	Pentecostals	17 (6.9)	4.2–10.5
	Others	5 (2.0)	0.7–4.4

### The Percentage Score for General Knowledge, Attitude, and Practices Among Rural Market Vendors

**Figure 1:**
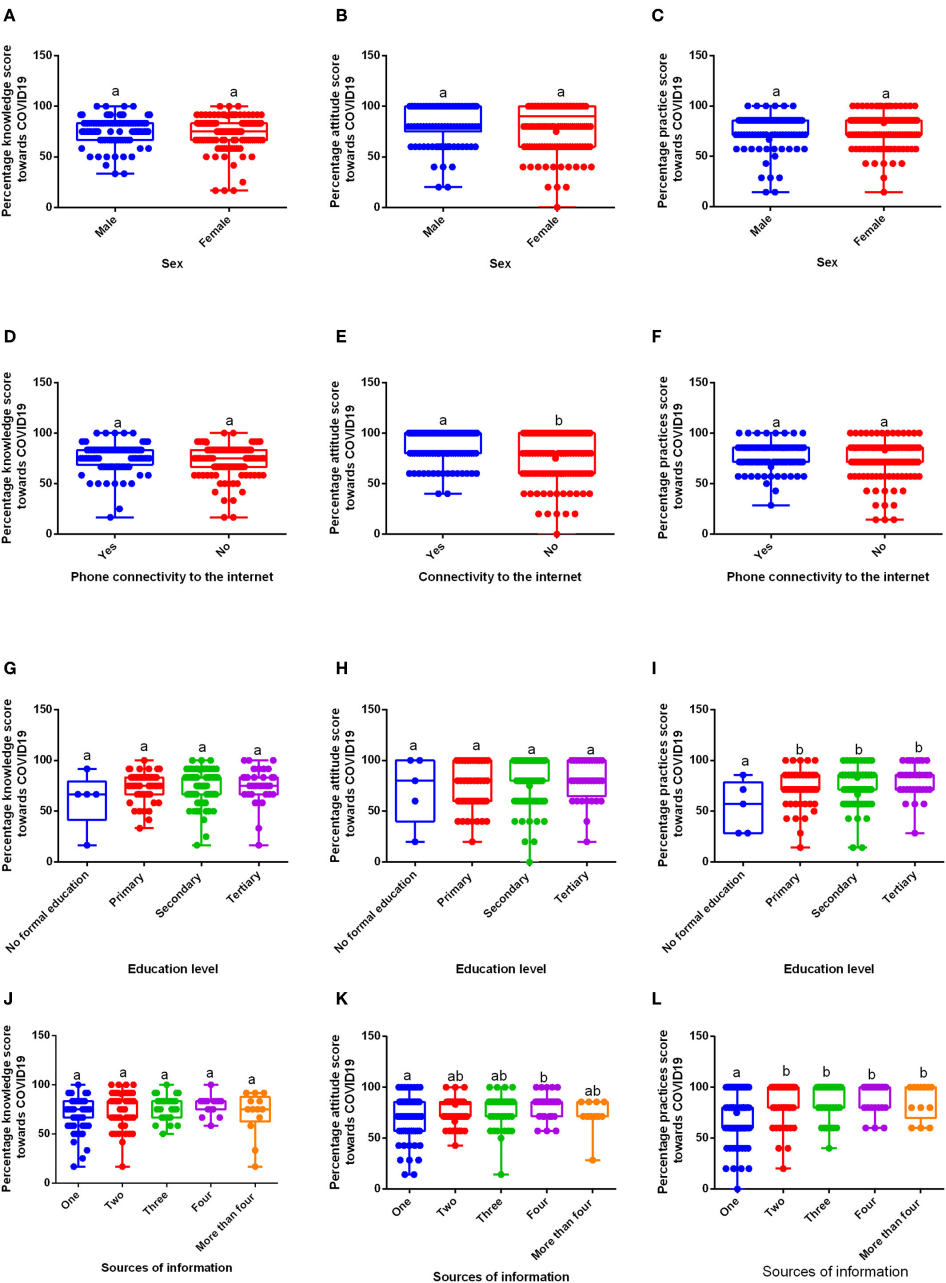
Percentage knowledge, attitude, and practice scores toward COVID-19 in the rural market vendors (*n*= 248). **(A–C)** Knowledge, Attitude, and Practice scores, respectively, in males and females; **(D–F)** Percentage knowledge, attitude, and practice scores, respectively, in relation to phone connectivity to the internet; **(G–I)** Percentage knowledge, attitude and practice scores in relation to different levels of education; **(J–L)** Percentage knowledge, attitude, and practice scores in relation to multiple sources of information. a and b show a significant difference at *p* ≤ 0.05.

The general percentage score was moderate for knowledge (75.57%) and practices (76.50%), but high for attitude (82.6%). The percentage score for knowledge and practice for males (76.41 and 75.10%, respectively) and females (75.05 and 77.35%, respectively) were moderate, however, a high score was observed for attitude among them; males (83.19%) and females (82.18%). The percentage score among those having their phones connected and those not having connected to the internet on knowledge (77.00 and 74.53%, respectively) and practices (78.49 and 75.08%, respectively) were moderate. However, there was a significant difference (*P* = 0.0055) in the percentage score for attitude among those connected (86.92%) and those not connected (79.41%) to the internet. The percentage score for practices was significantly different (*P* = 0.0058) among individuals with no formal education (54.29%) and those with primary (75.24%), secondary (77.03%), and tertiary (80.16%) levels of education. The percentage score for knowledge among those with one, two, three, and more than four sources of information (72.81, 75.88, 78.0, and 70.52%, respectively) was moderate. Nonetheless, a high score was reported for vendors with four sources of information (80.03%), although not significantly different. The percentage score for attitude was significantly different (*P* = 0.0358) among those with one source (73.04%) and those with four (82.68%) sources of information. The percentage score for practice was significantly different (*P* < 0.0001) between those with one source (69.19%) and those with two (89.07%), three (87.23%), four (92.12%), and more than four (86.15%) sources of information (Figure 1).

### Correlation Between Knowledge, Attitude, and Practices Among Rural Market Vendors

Correlation between knowledge on COVID-19, attitude, and practices toward COVID-19 among rural market vendors in western Uganda is presented in Table 2. There was a strong positive correlation between attitude and practices (*r* = 0.17, *p* = 0.007), as well as knowledge with practices (*r* = 0.29, *p* < 0.001).

**Table 2:** Correlation between knowledge, attitude, and practices toward COVID-19 among rural market vendors in western Uganda (*n* = 248).

	**Attitude**	**Practices**	**Knowledge**
Attitude	_	_	_
Practices	0.170^*^	_	_
Knowledge	0.109	0.291^*^	_

* *is significant at the 0.05 level. All r values are Spearman's correlation coefficients*.

### Relationship Between Gender and Level of Education, Sources of Information, Internet Connectivity, and Knowledge on COVID-19

The majority of the people 134/248 (54.0%) had attained a secondary level of education and were almost two times more than those who had no formal education (OR= 1.89). The majority of the people in the population 144/248 (58.1%) did not have their phones connected to the internet and were twice more than those whose phones were connected to the internet with significant differences between them (OR = 1.96, 95%CI: 1.16–3.31, *P* = 0.01). The majority of people 80/248 (32.3%) received their information regarding COVID-19 from one source and almost two times less than those who got information from four sources of information (OR = 1.55). Majority of the people 194/248 (78.2%) believe that elderly people, people with underlying chronic illnesses and the obese are more likely to develop severe cases of the infection and these are twice more than those who believe otherwise, with a significant difference between them (OR = 1.79, 95%CI: 0.93–3.54, *P* = 0.01). The majority of people 233/248 (94.0%) agreed, that the SARS CoV-2 spreads via respiratory droplets of infected individuals, and these are four times more than those who disagree but with no significant difference between them (OR = 4.24, 95%CI: 0.62–2.21, *P* = 0.05). (Table 3).

**Table 3:** Relationship between gender and level of education, sources of information, internet connectivity and knowledge on COVID-19.

		**GENDER**		**Total**	**OR**	**95%CI**	***P*-value**
		**Male**	**Female**				
Educational status	No formal education	1 (1.1)	4 (2.6)	5 (2.0)	1		0.129
	Primary level	33 (35.1)	40 (26.0)	73 (29.4)	3.30		0.58
	Secondary level	43 (45.7)	91 (59.1)	134 (54.0)	1.89		0.91
	Tertiary level	17 (18.1)	19 (12.3)	36 (14.5)	3.58		0.55
**Total**		**94 (100.0)**	**154 (100.0)**	**248 (100.0)**			
Phone internet connectivity	Yes	49 (52.1%)	55 (35.7)	104 (41.9)	1.96	1.16 to 3.31	0.01
	No	45 (47.9)	99 (64.3)	144 (58.1)			
**Total**		**94 (100.0%)**	**154 (100.0)**	**248 (100.0)**			
MultipleSource	one source	28 (29.8)	52 (33.8)	80 (32.3)	1		0.758
	two sources	26 (27.7)	49 (31.8)	75 (30.2)	0.99		0.002
	three sources	20 (21.3)	27 (17.5)	47 (19.0)	1.38		0.72
	four sources	15 (16.0)	18 (11.7)	33 (13.3)	1.55		1.07
	five and above	5 (5.3)	8 (5.2)	13 (5.2)	1.16		0.06
**Total**		**94 (100.0)**	**154 (100.0)**	**248 (100.0)**			
Multiple signs	One symptom	18 (19.1)	31 (20.1)	49 (19.8)	1		0.010
	Two Symptoms	43 (45.7)	82 (53.2)	125 (50.4)	0.90		0.10
	Three Symptoms	15 (16.0)	33 (21.4)	48 (19.4)	0.78		0.97
	Four Symptoms	12 (12.8)	7 (4.5)	19 (7.7)	2.95		0.18
	No idea	6 (6.4)	1 (.6)	7 (2.8)	10.33		0.05
**Total**		**94 (100.0)**	**154 (100.0)**	**248 (100.0)**			
There is no effective cure for COVID-2019 at the moment.	Yes	67 (71.3)	101 (65.6)	168 (67.7)	1.30	0.75 to 2.29	0.37
	No	27 (28.7)	53 (34.4)	80 (32.3)			
**Total**		**94 (100.0)**	**154 (100.0)**	**248 (100.0)**			
Early identification of symptoms and supportive care can help most patients recover from the infection	Yes	85 (90.4)	142 (92.2)	227 (91.5)	0.84	0.46 to 1.56	0.59
	No	9 (9.6)	12 (7.8)	21 (8.5)			
**Total**		**94 (100.0)**	**154 (100.0)**	**248 (100.0)**			
Not all persons with COVID-2019 will develop into severe cases.	Yes	71 (75.5)	121 (78.6)	192 (77.4)	0.84	0.46 to 1.56	0.59
	No	23 (24.5)	33 (21.4)	56 (22.6)			
**Total**		**94 (100.0)**	**154 (100.0)**	**248 (100.0)**			
Elderly people, people with underlying chronic illnesses and obese are more likely to develop severe cases	Yes	79 (84.0)	115 (74.7)	194 (78.2)	1.79	0.93 to 3.54	0.01
	No	15 (16.0)	39 (25.3)	54 (21.8)			
**Total**		**94 (100.0)**	**154 (100.0)**	**248 (100.0)**			
Eating or contacting wild animals would result in the infection by the COVID-19 virus	Yes	61 (64.9)	93 (60.4)	154 (62.1)	1.21	0.71 to 2.08	0.46
	No	33 (35.1)	61 (39.6)	94 (37.9)			
**Total**		**94 (100.0)**	**154 (100.0)**	**248 (100.0)**			
Persons with COVID-2019 cannot transmit the virus to others when a fever is not present	Yes	65 (69.1)	100 (64.9)	165 (66.5)	1.21	0.70 to 2.11	0.54
	No	29 (30.9)	54 (35.1)	83 (33.5)			
**Total**		**94 (100.0)**	**154 (100.0)**	**248 (100.0)**			
The COVID-19 virus spreads via respiratory droplets of infected individuals	Yes	92 (97.9)	141 (91.6)	233 (94.0)	4.24	0.92 to 39.40	0.05
	No	2 (2.1)	13 (8.4)	15 (6.0)			
**Total**		**94 (100.0)**	**154 (100.0)**	**248 (100.0)**			
Ordinary residents can wear general medical masks to prevent the infection by the COVID-19 virus	Yes	75 (79.8)	119 (77.3)	194 (78.2)	1.16	0.62 to 2.21	0.69
	No	19 (20.2)	35 (22.7)	54 (21.8)			
**Total**		**94 (100.0)**	**154 (100.0)**	**248 (100.0)**			
It is not necessary for children and young adults to take measures to prevent the infection by the COVID-19 virus	Yes	58 (61.7)	90 (58.4)	148 (59.7)	1.15	0.68 to 1.95	0.64
	No	36 (38.3)	64 (41.6)	100 (40.3)			
**Total**		**94 (100)**	**154 (100)**	**248 (100)**			
To prevent the infection by covid 19, individuals should avoid going to crowded areas	Yes	89 (94.7)	146 (94.8)	235 (94.8)	0.98	0.31 to 3.37	0.89
	No	5 (5.3)	8 (5.2)	13 (5.2)			
**Total**		**94 (100.0)**	**154 (100.0)**	**248 (100.0)**			
Isolation and treatment of people who are infected with the COVID-19 virus are effective ways to reduce the spread of the virus	Yes	92 (97.9)	144 (93.5)	236 (95.2)	3.19	0.66 to 30.52	0.14
	No	2 (2.1)	10 (6.5)	12 (4.8)			
**Total**		**94 (100.0)**	**154 (100.0)**	**248 (100.0)**			
People who have contact with someone infected with the COVID-19 virus should be immediately isolated in a proper place. In general, the observation period is 14 days	Yes	86 (91.5)	147 (95.5)	233 (94.0)	0.51	0.17 to 1.51	0.22
	No	8 (8.5)	7 (4.5)	15 (6.0)			
**Total**		**94 (100.0)**	**154 (100.0)**	**248 (100.0)**			

### Relationship Between Internet Connectivity and Knowledge on COVID-19

The majority 194/248 (78.2%) of our respondents agreed that elderly people, people with underlying chronic illnesses and obese are more likely to develop severe cases, out of which most 114/194 (58.8%) did not have internet connectivity on their phones, although with no difference (OR = 0.88; 95% CI = 0.48–1.63; *P* = 0.70). The majority 154/248 (62.1%) of our respondents agreed that eating or contacting wild animals would result in the infection by the SARS CoV-2, out of the proportion having their phones connected to the internet equal those who do not 77/154 (50.0%). Respondents with internet connectivity on their phones who agreed that eating or contacting wild animals would result in the infection by the SARS CoV-2 were two times greater than those who do not agree (OR = 2.48; 95% CI = 1.44–4.32; *P* = 0.001). The majority 165/248 (66.5%) of our respondents agreed that persons with COVID-2019 cannot transmit the virus to others when a fever is not present, out of which most 96/165 (58.2%) did not have internet connectivity on their phones, although with no difference (OR = 0.99, 95% CI = 0.58–1.69; *P* = 0.95).

The majority 233/248 (94.0%) of our respondents agreed that the SARS CoV-2 spreads via respiratory droplets of infected individuals, out of which most 138/233 (59.2%) did not have internet connectivity on their phones, although with no difference (OR = 0.46; 95% CI = 0.15–1.35; *P* = 0.14). The majority 194/248 (78.2%) of our respondents agreed that ordinary residents can wear general medical masks to prevent the infection by the SARS CoV-2, out of which most 111/194 (57.2%) did not have internet connectivity on their phones, although with no difference (OR = 1.18; 95% CI = 0.63–2.20; *P* = 0.59). The majority 148/248 (59.7%) of our respondents agreed that children and young adults don't need to take measures to prevent the infection by the SARS CoV-2, out of which most 81/148 (54.7%) did not have internet connectivity on their phones, although with no difference (OR = 1.41; 95% CI = 0.84–2.38; *P* = 0.22).

The majority 235/248 (94.8%) of our respondents agreed that to prevent the infection by COVID-19, individuals should avoid going to crowded places, out of which most 135/235 (57.4%) did not have internet connectivity on their phones. Respondents with internet connectivity on their phones who agreed that to prevent the infection by COVID-19, individuals should avoid going to crowded places were 2 times greater (OR = 1.67; 95% CI = 0.45–7.60; *P* = 0.57). The majority 236/248 (95.2%) of our respondents agreed that isolation and treatment of people who are infected with the SARS CoV-2are effective ways to reduce the spread of the virus, out of which most 135/236 (57.2%) did not have internet connectivity on their phones. Respondents with internet connectivity on their phones who agreed that isolation and treatment of people who are infected with the SARS CoV-2 are effective ways to reduce the spread of the virus were 2 times greater (OR = 2.24; 95% CI = 0.54–13.17; *P* = 0.37) (Table 4).

**Table 4:** Relationship between phone internet connectivity and knowledge on COVID-19.

		**Phone internet connectivity**		**Total**			
		**Yes**	**No**		**OR**	**CI**	***P*-value**
Elderly people, people with underlying chronic illnesses and obese are more likely to develop severe cases	Yes	80 (41.2)	114 (58.8)	194 (100.0)	0.88	0.48–1.63	0.70
	No	24 (44.4)	30 (55.6)	54 (100.0)			
Eating or contacting wild animals would result in the infection by the COVID−19 virus	Yes	77 (50.0)	77 (50.0)	154 (100.0)	2.48	1.44–4.32	0.001
	No	27 (28.7)	67 (71.3)	94 (100.0)			
Persons with COVID-2019 cannot transmit the virus to others when a fever is not present	Yes	69 (41.8)	96 (58.2)	165 (100.0)	0.99	0.58–1.69	0.95
	No	35 (42.2)	48 (57.8)	83 (100.0)			
The COVID-19 virus spreads via respiratory droplets of infected individuals	Yes	95 (40.8)	138 (59.2)	233 (100.0)	0.46	0.15–1.35	0.14
	No	9 (60.0)	6 (40.0)	15 (100.0)			
Ordinary residents can wear general medical masks to prevent the infection by the COVID-19 virus	Yes	83 (42.8)	111 (57.2)	194 (100.0)	1.18	0.63–2.20	0.59
	No	21 (38.9)	33 (61.1)	54 (100.0)			
It is not necessary for children and young adults to take measures to prevent the infection by the COVID-19 virus	Yes	67 (45.3)	81 (54.7)	148 (100.0)	1.41	0.84–2.38	0.22
	No	37 (37.0)	63 (63.0)	100 (100.0)			
To prevent the infection by COVID-19, individuals should avoid going to crowded places	Yes	100 (42.6)	135 (57.4)	235 (100.0)	1.67	0.45–7.60	0.57
	No	4 (30.8)	9 (69.2)	13 (100.0)			
Isolation and treatment of people who are infected with the COVID-19 virus are effective ways to reduce the spread of the virus	Yes	101 (42.8)	135 (57.2)	236 (100.0)	2.24	0.54–13.17	0.37
	No	3 (25.0)	9 (75.0)	12 (100.0)			
People who have contact with someone infected with the COVID-19 virus should be immediately isolated in a proper place. In general, the observation period is 14 days	Yes	98 (42.1)	135 (57.9)	233 (100.0)	1.09	0.37–3.39	0.9
	No	6 (40.0)	9 (60.0)	15 (100.0)			
Sample				248			

### Sources of Information and Knowledge on COVID-19 Among Market Vendors

The majority 125/248 (50.4%) of our respondents knew two symptoms of COVID-19, out of which most 39/125 (48.80%) got their information from one source of information. The majority 192/248 (77.1%) of our respondents agreed that not all persons with COVID-2019 will develop to severe cases, out of which most 60/192 (31.3%) had one source of information. The majority 194/248 (78.2%) of our respondents agreed that Elderly people, people with underlying chronic illnesses and obese are more likely to develop severe cases, out of which most 62/194 (32.0%) had one source of information on COVID-19. The majority 154/248 (62.1%) of our respondents agreed that Eating or contacting wild animals would result in the infection by the SARS CoV-2, out of which most had two sources 58/154 (37.7%) of information on COVID-19. The majority 165/248 (66.5%) of our respondents agreed that persons with COVID-2019 cannot transmit the virus to others when a fever is not present, out of which most had two sources 55/165 (33.3%) of information on COVID-19. The majority 233/248 (94.0%) of our respondents agreed that the SARS CoV-2spreads via respiratory droplets of infected individuals, out of which most had one source 75/233 (32.2%) of information on COVID-19.

The majority 194/248 (78.2%) of our respondents agreed that ordinary residents can wear general medical masks to prevent the infection by the SARS CoV-2, out of which most had one source 63/194 (32.5%) of information on COVID-19. The majority 148/248 (59.7%) of our respondents agreed that children and young adults don't need to take measures to prevent the infection by the SARS CoV-2, out of which most had two sources 45/148 (30.4) of information on COVID 19. The majority 235/248 (94.8%) of our respondents agreed that to prevent the infection by COVID-19, individuals should avoid going to crowded places, out of which most had one source 74/235 (31.4%) of information on COVID 19. The majority 236/248 (95.2%) of our respondents agreed that isolation and treatment of people who are infected with the SARS CoV-2are effective ways to reduce the spread of the virus, out of which most had one source of information on COVID 19 74/236 (31.4%). The majority 233/248 (94.0%) of our respondents agreed that people who have contact with someone infected with the SARS CoV-2 should be immediately isolated in a proper place. In general, the observation period is 14 days, out of which most had one 74/233 (31.8%) source of information on COVID 19 (Table 5).

**Table 5:** Source of information, education level and knowledge on COVID 19 among market vendors.

		**Multiple sources**					**Total**	**Educational status**				**Total**
		**One source**	**Two sources**	**Three sources**	**Four sources**	**Five and above**		**No formal education**	**Primary level**	**Secondary level**	**Tertiary level**	
Multiple symptoms	One Symptom	28 (35.00)	17 (22.70)	3 (6.40)	1 (3.00)	0 (0.00)	**49**	2 (40.0)	18 (24.7)	23 (17.2)	6 (16.7)	**49**
	Two Symptoms	39 (48.80)	32 (42.70)	27 (57.40)	20 (60.60)	7 (53.80)	**125**	1 (20.0)	37 (50.7)	73 (54.5)	14 (38.9)	**125**
	Three Symptoms	10 (12.50)	21 (28.00)	12 (25.50)	5 (15.20)	0 (0.00)	**48**	1 (20.0)	15 (20.5)	27 (20.1)	5 (13.9)	**48**
	Four Symptoms	1 (1.20)	4 (5.30)	5 (10.60)	6 (18.20)	3 (23.10)	**19**	1 (20.0)	0 (0.0)	9 (6.7)	9 (25.0)	**19**
	No idea	2 (2.50)	1 (1.30)	0 (0.00)	1 (3.00)	3 (23.10)	**7**	0 (0.0)	3 (4.1)	2 (1.5)	2 (5.6)	**7**
Not all persons with COVID-2019 will develop to severe cases.	Yes	59 (30.7)	60 (31.3)	35 (18.2)	29 (15.1)	9 (4.7)	**192**	1 (0.5)	58 (30.2)	112 (58.3)	21 (10.9)	**192**
	No	21 (37.5)	15 (26.8)	12 (21.4)	4 (7.1)	4 (7.1)	**56**	47 (7.1)	15 (26.8)	22 (39.3)	15 (26.8)	**56**
Elderly people, people with underlying chronic illnesses and obese are more likely to develop severe cases	Yes	62 (32.0)	55 (28.4)	39 (20.1)	29 (14.9)	9 (4.6)	**194**	2 (1.0)	58 (29.9)	107 (55.2)	27 (13.9)	**194**
	No	18 (33.3)	20 (37.0)	8 (14.8)	4 (7.4)	4 (7.4)	**54**	3 (5.6)	15 (27.8)	27 (50.0)	9 (16.7)	**54**
Eating or contacting wild animals would result in the infection by the COVID-19 virus	Yes	33 (21.4)	58 (37.7)	31 (20.1)	24 (15.6)	8 (5.2)	**154**	4 (2.6)	42 (27.3)	82 (53.2)	26 (16.9)	**154**
	No	47 (50.0)	17 (18.1)	16 (17.0)	9 (9.6)	5 (5.3)	**94**	1 (1.1)	31 (33.0)	52 (55.3)	10 (10.6)	**94**
Persons with COVID-2019 cannot transmit the virus to others when a fever is not present	Yes	50 (30.3)	55 (33.3)	31 (18.8)	23 (13.9)	6 (3.6)	**165**	1 (0.6)	51 (30.9)	96 (58.2)	17 (10.3)	**165**
	No	30 (36.1)	20 (24.1)	16 (19.3)	10 (12.0)	7 (8.4)	**83**	4 (4.8)	22 (26.5)	38 (45.8)	19 (22.9)	**83**
The COVID-19 virus spreads via respiratory droplets of infected individuals	Yes	75 (32.2)	70 (30.0)	44 (18.9)	32 (13.7)	12 (5.2)	**233**	4 (1.7)	70 (30.0)	129 (55.4)	30 (12.9)	**233**
	No	5 (33.3)	5 (33.3)	3 (20.0)	1 (6.7)	1 (6.7)	**15**	1 (6.7)	3 (20.0)	5 (33.3)	6 (40.0)	**15**
Ordinary residents can wear general medical masks to prevent the infection by the COVID-19 virus	Yes	63 (32.5)	56 (28.9)	38 (19.6)	29 (14.9)	8 (4.1)	**194**	2 (1.0)	56 (28.9)	108 (55.7)	28 (14.4)	**194**
	No	17 (31.5)	19 (35.2)	9 (16.7)	4 (7.4)	5 (9.3)	**54**	3 (5.6)	17 (31.5)	26 (48.1)	8 (14.8)	**54**
It is not necessary for children and young adults to take measures to prevent the infection by the COVID-19 virus	Yes	39 (26.4)	45 (30.4)	30 (20.3)	25 (16.9)	9 (6.1)	**148**	1 (0.7)	41 (27.7)	89 (60.1)	17 (11.5)	**148**
	No	41 (41.0)	30 (30.0)	17 (17.0)	8 (8.0)	4 (4.0)	**100**	4 (4.0)	32 (32.0)	45 (45.0)	19 (19.0)	**100**
To prevent the infection by COVID-19, individuals should avoid going to crowded places	Yes	71 (30.2)	72 (30.6)	47 (20.0)	33 (14.0)	12 (5.1)	**235**	4 (1.7)	66 (28.1)	129 (54.9)	36 (15.3)	**235**
	No	9 (69.2)	3 (23.1)	0 (0.0)	0 (0.0)	1 (7.7)	**13**	1 (7.7)	7 (53.8)	5 (38.5)	0 (0.0)	**13**
Isolation and treatment of people who are infected with the COVID-19 virus are effective ways to reduce the spread of the virus	Yes	74 (31.4)	71 (30.1)	46 (19.5)	33 (14.0)	12 (5.1)	**236**	4 (1.7)	71 (30.1)	126 (53.4)	35 (14.8)	**236**
	No	6 (50.0)	4 (33.3)	1 (8.3)	0 (0.0)	1 (8.3)	**12**	1 (8.3)	2 (16.7)	8 (66.7)	1 (8.3)	**12**
People who have contact with someone infected with the COVID-19 virus should be immediately isolated in a proper place. In general, the observation period is 14 days	Yes	74 (31.8)	71 (30.5)	45 (19.3)	32 (13.7)	11 (4.7)	**233**	4 (1.7)	69 (29.6)	126 (54.1)	34 (14.6)	**233**
	No	6 (40.0)	4 (26.7)	2 (13.3)	1 (6.7)	2 (13.3)	**15**	1 (6.7)	4 (26.7)	8 (53.3)	2 (13.3)	**15**

### Education Level and Knowledge on COVID-19 Among Market Vendors

The majority 125/248 (50.4%) of our respondents knew two symptoms of COVID 19, out of which most 73/125 (54.5%) had secondary education. The majority 192/248 (77.4%) of our respondents agreed that not all persons with COVID-2019 will develop to severe cases, out of which most 112/192 (58.3%) had secondary education. The majority 194/248 (78.2%) of our respondents agreed that Elderly people, people with underlying chronic illnesses, and obese are more likely to develop severe cases, out of which most 107/194 (55.2%) had secondary education. The majority 154/248 (62.1%) of our respondents agreed that Eating or contacting wild animals would result in the infection by the SARS CoV-2, out of which most 82/154 (53.2%) had secondary education.

The majority 165/248 (66.5%) of our respondents agreed that persons with COVID-2019 cannot transmit the virus to others when a fever is not present, out of which most 96/165 (58.2%) had secondary education. The majority 233/248 (94.0%) of our respondents agreed that the SARS CoV-2spreads via respiratory droplets of infected individuals, out of which most 129/233 (55.4%) had secondary education. The majority 194/248 (78.2%) of our respondents agreed that ordinary residents can wear general medical masks to prevent the infection by the SARS CoV-2, out of which most 108/194 (55.7%) had secondary education.

The majority 148/248 (59.7%) of our respondents agreed that children and young adults don't need to take measures to prevent the infection by the SARS CoV-2, out of which most 89/148 (60.1%) had secondary education. The majority 235/248 (94.8%) of our respondents agreed that to prevent the infection by COVID19, individuals should avoid going to crowded places, out of which most 129/235 (54.9%) had secondary education. The majority 236/248 (95.2%) of our respondents agreed that isolation and treatment of people who are infected with the SARS CoV-2are effective ways to reduce the spread of the virus, out of which most 126/236 (53.4%) had secondary education. The majority 233/248 (94.0%) of our respondents agreed that people who have contact with someone infected with the SARS CoV-2should be immediately isolated in a proper place. In general, the observation period is 14 days, out of which most 126/233 (54.1%) had secondary education (Table 5).

## Discussion

The majority of market vendors in Bushenyi were female youths between the ages of 20–30 years and they had attained some level of formal education (98%) with 14.5% having had a professional certificate. These sociodemographic characteristics are typical of market vendors in Uganda (9, 14) except that the biggest proportion of our market vendors (98%) had formal education compared to those in previous studies 60–80% with only 6.6% of these having had a professional course. A recent study in Africa confirmed the role of gender differences in curbing epidemics in informal settlements which cannot be overlooked (15) during the control COVID-19. Furthermore, women and girls are at increased risk during epidemics due to various reasons like being responsible for caring for children and the elderly and being the majority of the health care workers in Africa (15). This is a good indicator because education level and professional training are very instrumental in shaping people's knowledge, attitudes, and practices regarding COVID-19 (29). It is therefore vital to focus on increasing the education level of the community putting special emphasis on the females when creating preventive and mitigation measures for COVID-19 among rural market vendors and thus reducing their risk of infection (15).

Knowledge is crucial in shaping people's behavior and practices especially during any disease outbreak (17). There was a positive correlation between knowledge with practices, and attitude with practices. This was in line with the general notion that adequate knowledge is often associated with good attitudes and practices (18). This is because knowledge level is linked with panic emotion among most populations, which in turn influences their attitude and practices toward COVID-19 (18). Most of the vendors had adequate knowledge on COVID-19 and this was a novel finding in the study since market vendors are often associated with less knowledgeable and careless behavior toward public health emergencies (9). The adequate knowledge about COVID-19 observed among rural market vendors was attributed to the government's engagement of the local communities, trained health workers, different security agencies to enforce those outlined measures (25). The reported moderate level of COVID-19 awareness could also be attributed to them having a formal education, and awareness campaigns carried out by the government in the electronic media. This concurs with recent studies that showed that a person's level of education is very instrumental in shaping people's knowledge, attitudes, and practices regarding COVID-19 (15, 29). Findings in the current study are in agreement with thee observations that the vendors' percentage score for practice regarding COVID-19 was significantly low (poor practices) in those who had no formal education compared to those who had formal education. This is because people with no formal education are more likely to engage in risky behaviors than those with formal education (34). The less severe situation in the COVID 19 outbreak in Uganda could also be a possible reason for some pockets of recorded poor practices among those with no formal education (4). In general, the reported moderate score for practices among the rural market vendors, aside having good knowledge about COVID 19 and some level of formal education, could be linked with the fear of punishment by the law enforcement agents for flaunting the government directives especially in public places such as the washing of hands, as soaps and water are been stationed at strategic points to facilitate compliance. More females than males knew these facts about COVID 19 because more females participated in the study than males but there was no significant difference among them concurring with a recent COVID-19 KAP study in Kampala where there were no gender differences in the study (35).

Even though the majority of the vendors had adequate knowledge regarding the eight (8) COVID19, there was a deviation in knowledge among them on the following COVID-19 facts: Symptoms; elderly people, people with underlying chronic illnesses and the obese are more likely to develop severe cases of the infection; SARS CoV-2spreads via respiratory droplets of infected individuals; isolation and treatment of people who are infected with the SARS CoV-2are effective ways to reduce the spread of the virus, and could be attributed to the fact that the illness is a new one (36, 37). This fact is a pointer that the government should not relax its efforts on publicity on COVID 19, and strengthen the public's alertness to COVID-19, inform the public on the importance of protecting themselves with enough precautionary measures (38).

Misinformation among market vendors can be more disastrous and may act as a tool to worsen the epidemiologic characteristics of the pandemic since a rise in COVID 19 cases among them will expose the local community to an increased risk of infection (21, 22). Given the present challenge, the major sources of information regarding the COVID-19 pandemic could either guarantee success or compromise the fight, especially among market vendors who are mostly rural settlers (23). The majority of rural market vendors depended on one source of information to obtain knowledge on COVID-19. Although there was no significant difference in the knowledge score between those with one source of information and those with two, three, four, and more sources of information, although all categories had adequate knowledge and good practices toward COVID−19. This could be attributed to higher levels and the proportion of educated people in our study population who are actively using more information-seeking behaviors without necessarily relying on their counterparts (39–41). The use of radio was a universal source among rural market vendors with one and multiple sources of information, this finding contradicts the findings of Ikoja-Odongo (24) who indicated that personal experiences and friends are the major sources of information with social media and radios only coming in at third among Ugandans belonging to the informal sectors.

Phone internet connectivity did not affect the level of knowledge on COVID-19 among rural market vendors in the present studies, this is supported by the fact that other studies in Uganda don't mention internet and social media as the major sources of information from which health information is obtained among market people (24). Even though there are no significant differences in the knowledge regarding the pandemics among vendors whose phones were connected and those not connected to the internet, the former were more knowledgeable about most of the following COVID-19 facts: eating or contacting wild animals would result in the infection by the SARS CoV-2, it is not necessary for children and young adults to take measures to prevent the infection by the SARS CoV-2, to prevent the infection by COVID-19, individuals should avoid going to crowded places, isolation, and treatment of people who are infected with the SARS CoV-2 are effective ways to reduce the spread of the virus. This implies that, although phone internet connectivity was not popular among rural market vendors, it plays a vital role in improving people's awareness on COVID-19 and internet tools such as social media should be incorporated in the quest to halt the rapid spread of COVID-19 among rural dwellers (26), because the majority of the rural market vendors in Uganda are youths. It is also important to note that market vendors whose phones were connected to the internet had higher percentage score for attitude (positive attitudes) toward COVID-19, settling the role of internet tools and the need for their incorporation in the prevention, management, and prognosis of COVID-19 not only in Uganda but globally (25–27).

## Conclusion

Where and how the rural market vendors get their information and education level are vital in breaking COVID 19 infection circle in line with WHO guidelines. Therefore, sources of information and education level played a key role in molding their knowledge and practices. However, the level of knowledge on COVID 19 among our respondents was not linked with phone internet connectivity. To guarantee successful containment of SARS CoV-2, the government needs to enact a more robust strategy by paying attention to educating rural market vendors, as well as the rest of community members, and adequate utilization of affordable, reliable, and more effective sources of information to reach the rural people.

## Data Availability Statement

The datasets presented in this study can be found in online repositories. The names of the repository/repositories and accession number(s) can be found below: https://figshare.com/s/b024cdd4e37fbdd0d399.

## Ethics Statement

Expediated ethical approval from Kampala International Ethical Review Board was acquired and registered as Nr.UG-REC-023/201914. Written, informed consent was obtained from the study participants while in minors, this was first acquired from their legal guardians and then individually from each participant who was a minor for the publication of any potentially identifiable images or data included in this article. Written informed consent to participate in this study was provided by the participants' legal guardian/next of kin.

## Author Contributions

IU, FS, and KK conceptualized the study. IU, FS, RS, AL, VA, and KK designed the study. IU, FS, AL, JOA, EK, JTA, OO, EA, IE, AA, RM, and VN collected the data. AL, VA, JOA, EK, JTA, OO, EA, IE, AA, RM, and VN conducted data analysis while AL, VA, JOA, EK, JTA, OO, EA, IE, SK, AA, RM, and VN interpreted the data. IU and FS drafted initial version, RS, AL, VA, JOA, EK, JTA, OO, EA, IE, SK, AA, RM, VN, and KK revising it critically for important intellectual content approved final approval of the version to be published. All authors remain in agreement to be accountable for all aspects of the work.

## Conflict of Interest

The authors declare that the research was conducted in the absence of any commercial or financial relationships that could be construed as a potential conflict of interest.

## References

[1] Wu YC Chen CS Chan Y. J. The outbreak of COVID-19: an overview. J Chinese Med Assoc. (2020) 83:3–217. 10.1097/JCMA.0000000000000270 32134861 PMC7153464

[2] Guo YR Cao QD Hong ZS Tan YY Chen SD Jin HJ & Yan Y. The origin, transmission and clinical therapies on coronavirus disease 2019 (COVID-19) outbreak–an update on the status. Milit Med Res. (2020) 7:1–10. 10.1186/s40779-020-00240-0 PMC7068984 32169119

[3] EAC EAC Secretariat urges Partner States to Prepare Economic Recovery Plans for the Time After COVID-19. Published by EAC. (2020). Available online at: https://eac.int/press-releases/147-health/1711-eac-secretariat-urges-partnerstates-to-prepare-economic-recovery-plans-for-the-time-after-covid-19 (accessed May 10, 2020).

[4] Agence F Uganda Orders Two-Week Lockdown to Contain Coronavirus. Courthouse News Service on March. (2020) Available online at: https://www.courthousenews.com/uganda-orders-two-weeklockdown-to-contain-coronavirus/ (accessed May 8, 2020).

[5] The Economic Times Coronavirus: Will the Lockdown Create a Supply Shock That. India simply can't afford? The Economic Times(2020). Available online at: https://economictimes.indiatimes.com/news/politics-and-nation/india-stares-at-acrippling-supply-crisis/articleshow/74801350.cms (accessed May 10, 2020).

[6] Opara-Martins J Sahandi Tian RF Critical analysis of vendor lock-in and its impact on cloud computing migration: a business perspective. J Cloud Comp. (2016) 5:4 10.1186/s13677-016-0054-z

[7] Karsten N In Africa, Social Distancing Is a Privilege Few Can Afford. (2020). Available online at: https://www.aljazeera.com/indepth/opinion/africa-social-distancing-privilege-afford200318151958670.html (accessed May 10, 2020).

[8] Pena S Regulating informal markets: informal commerce in Mexico City. Int J Sociol Soc Policy. (2000) 20:37–67. 10.1108/01443330010789223

[9] Muyanja C Nayiga L Brenda N & Nasinyama G Practices, knowledge and risk factors of street food vendors in Uganda. Food Control. (2011) 22:1551–8. 10.1016/j.foodcont.2011.01.016

[10] Ozili PK COVID-19 in Africa: Socioeconomic Impact, Policy Response and Opportunities. Policy Response and Opportunities. (2020) Available online at: https://mpra.ub.unimuenchen.de/99617/1/MPRA_paper_99617.pdf. 10.2139/ssrn.3574767

[11] Makinde T Problems of policy implementation in developing nations: The Nigerian experience. J Soc Sci. (2005) 11:63–69. 10.1080/09718923.2005.11892495

[12] Ministry of Health. National Guidelines for Management of Covid-19, Published by the Ministry of Health, Uganda. (2020). Available online at: https://www.health.go.ug/covid/document/national-guidelines-formanagement-of-covid-19/ (accessed May 11, 2020).

[13] Frank M Empowering Female Traders in East Africa Will Boost Growth – and Fight Poverty. Published by The Guardian (2020). Available online at: https://www.theguardian.com/global-development/2015/dec/15/empowering-femaletraders-east-africa-will-boost-growth-fight-poverty (accessed May 10, 2020).

[14] Phoebe KP Samuel MI Julius MM Jesca LN Socio-demographic characteristics of vendors and manufacturing practices of fresh unpasteurized fruit and vegetable juices in kampala, uganda. Int J Adv Res.(2019) 7:372–9. 10.21474/IJAR01/8337

[15] Austrian K Pinchoff J Tidwell JB White C Abuya T Kangwana B COVID-19 related knowledge, attitudes, practices and needs of households in informal settlements in Nairobi, Kenya. Bull World Health Organ. (2020). 10.2471/BLT.20.260281

[16] Bennett R Chepngeno-Langat G Evandrou M Falkingham J. Gender differentials and old age survival in the Nairobi slums, Kenya. Soc Sci. (2016) 163:107–116. 10.1016/j.socscimed.2016.07.002 27423067

[17] Abdulkareem SA Augustijn EW Filatova T Musial K Mustafa YT. Risk perception and behavioral change during epidemics: comparing models of individual and collective learning. PLoS ONE. (2020) 15:e0226483. 10.1371/journal.pone.0226483 31905206 PMC6944362

[18] Zhong BL Luo W Li HM Zhang QQ Liu XG Li WT. Knowledge, attitudes, and practices towards COVID-19 among Chinese residents during the rapid rise period of the COVID-19 outbreak: a quick online cross-sectional survey. Int J Biol Sci. (2020) 16:1745–52. 10.7150/ijbs.45221 32226294 PMC7098034

[19] Li Z Jin H Chen W Sun Z Jing L Zhao X. Influencing factors of knowledge, attitude, and practice regarding medical nutrition therapy in patients with diabetes: a national cross-sectional study in urban China. J Diabetes Res. (2017) 2017:8948452. 10.1155/2017/8948452 28948173 PMC5602617

[20] Caroline B Companies Fear Rise of Fake News and Social Media Rumours. Financial Time (2020). Available online at: https://www.ft.com/content/4241a2f6-e080-11e9-9743-db5a370481bc (accessed May 10, 2020).

[21] Jeanne S How to Help Your Favorite Small Businesses Survive the Coronavirus Crisis. CNN (2020). Available online at: https://edition.cnn.com/2020/03/15/success/small-businessescoronavirus/index.html (accessed May 11, 2020).

[22] Odoobo CB Fake news on coronavirus: Media should create ‘Fact Check' Corner. Daily Monitor (2020). Available online at: https://www.monitor.co.ug/OpEd/Commentary/Fake-news-coronavirus-Mediashould-create-Fact-Check-corner/689364-5506266-mbtl1e/index.html (accessed May 11, 2020).

[23] WHO Africa Uganda Uses Recent Outbreak Experience to Prepare for Coronavirus. WHO (2020). Available online at: https://www.afro.who.int/news/uganda-uses-recent-outbreakexperience-prepare-coronavirus (accessed May 11, 2020).

[24] Ikoja-Odongo R Insights into the information needs of women in the informal sector of Uganda. South Afri J Library Inform Sci.(2002) 68:39–52. 10.7553/68-1-765

[25] Geiger D Harborth L Mugyisha A Managing enduring public health emergencies such as COVID-19: lessons from Uganda Red Cross Society's Ebola virus disease response operation. BMJ Leader. (2020). 10.1136/leader-2020-000243

[26] Ronald O Social Media Platforms for Health Communication and Research in the Face of COVID-19 Pandemic: A Cross Sectional Survey in Uganda. (2020) Available online at: https://www.medrxiv.org/content/10.1101/2020.04.30.20086553v1

[27] Zhou X Snoswell CL Harding LE Bambling M Edirippulige S Bai X. The role of telehealth in reducing the mental health burden from COVID-19. Telemed J E-Health. (2020) 26:377–9. 10.1089/tmj.2020.0068 32202977

[28] CIPESA staff How Technology is Aiding the Covid-19 Fight in Africa. (2020). Available online at: https://cipesa.org/2020/03/how-technology-isaiding-the-covid-19-fight-in-africa/ (accessed May 7, 2020).

[29] Shi Y Wang J Yang Y Wang Z Wang G Hashimoto K. Knowledge and attitudes of medical staff in Chinese psychiatric hospitals regarding COVID-19. Brain Behav Immunity. (2020) 4:100064. 10.1016/j.bbih.2020.100064 32289123 PMC7138160

[30] Holakouie-Naieni K Ahmadvand A Raza O Assan A Elduma AH Jammeh A.. Assessing the knowledge, attitudes, and practices of students regarding ebola virus disease outbreak. Iran J Public Health. (2015) 44:1670–6. 26811818 PMC4724740

[31] WHO Key Messages and Actions for COVID-19 Prevention and Control in Schools. Published by WHO. (2020). Available online at: https://www.who.int/docs/default-source/coronaviruse/key-messages-and-actions-for-covid-19-prevention-and-control-in-schools-march-2020.pdf?sfvrsn=baf81d52_4 (accecced May 25, 2020).

[32] López-Goñi I Coronavirus: Ten Reasons Why You Ought Not to Panic. The conversation. (2020). Available online at: https://theconversation.com/coronavirus-ten-reasons-why-you-ought-not-to-panic-132941 (accecced May 26, 2020).

[33] Hui-Chin K Poh KB Ruzita AT Assessment of knowledge, attitude and practice towards whole grains among children aged 10 and 11 years in Kuala Lumpur, Malaysia. Int J Food Sci Nutr Diet. (2015) 04:171–7. 10.19070/2326-3350-1500032

[34] Swahn MH Buchongo P Kasirye R. Risky behaviors of youth living in the slums of kampala: a closer examination of youth participating in vocational training programs. Vuln Children Youth Studies. (2018) 13:276–90. 10.1080/17450128.2018.1489168 31452668 PMC6709978

[35] Olum R Chekwech G Wekha G Nassozi DR Bongomin F. Coronavirus disease-2019: knowledge, attitude, and practices of health care workers at makerere university teaching hospitals, Uganda. Front Public Health. (2020) 8:181. 10.3389/fpubh.2020.00181 32426320 PMC7204940

[36] WHO. ‘Report of the WHO-China Joint Mission on Coronavirus. Disease 2019 (COVID-19). WHO (2020). Available online at: https://www.who.int/docs/default-source/coronaviruse/who-china-joint-mission-on-covid-19-final-report.pdf (accessed May 8, 2020).

[37] Gayawan E Awe O Oseni BM Uzochukwu IC Adekunle AI Samuel G The spatio-temporal epidemic dynamics of COVID-19 outbreak in Africa. medRxiv. (2020) 10.1101/2020.04.21.20074435 PMC7506177 32873352

[38] Hu D Lou X Xu Z Meng N Xie Q Zhang M. More effective strategies are required to strengthen public awareness of COVID-19: Evidence from Google Trends. J Global Health. (2020) 10:010902. 10.7189/jogh.10.0101003 32373339 PMC7182392

[39] Limberg L Sundin O Teaching information seeking: relating information literacy education to theories of information behavior. Inf Res. (2006) 12:280.

[40] Ajiboye JO Tella A University undergraduate students' information. seeking behaviour: implications for quality in higher education in Africa. Turkish Online J Educ Technol. (2007) 6:40–52.

[41] Musoke D Boynton P Butler C Musoke MB. Health seeking behaviour and challenges in utilising health facilities in Wakiso district, Uganda. African Health Sci. (2014) 14:1046–55. 10.4314/ahs.v14i4.36 25834516 PMC4370086

