# Supplementary material for: Community Drivers Affecting Adherence to WHO Guidelines Against COVID-19 Amongst Rural Ugandan Market Vendors
Source: Front Public Health. 2020 Jul 3;8:340. doi: 10.3389/fpubh.2020.00340 (PMC7357280; doi:10.3389/fpubh.2020.00340)
Supplement: Supplementary file 1 [file Data_Sheet_1.docx]

Questionnaire on: Community drivers affecting adherence to WHO guidelines against COVID-19 amongst rural Ugandan market vendors

Age of respondent .....................................

**Section A**

This section contains questions on the demographic characteristics of the respondents**.**

**Table 1: Demographic characteristics Instruction:** Tick appropriately

| **S/N** | **Characteristics** | **Categories** | |
| --- | --- | --- | --- |
| 1 | Gender | Male  Female |  |
|  |  |  |  |
| 3 | Educational status | Primary level  Secondary level  Tertiary level  None of the above |  |
|  |  |  |  |
|  |  |  |  |
|  |  |  |  |
| 4 | Marital Status | Single  Married  Divorced  Widow |  |
|  |  |  |  |
|  |  |  |  |
|  |  |  |  |
| 5 | Religion | Protestants  Catholic  Adventist  Muslim  Pentecostals  Others |  |
|  |  |  |  |
|  |  |  |  |
|  |  |  |  |
|  |  |  |  |
|  |  |  |  |

The section contains questions on the knowledge of the respondents about COVID-19

**Instruction**: Tick appropriately

1. **The clinical symptoms of COVID-19 are;**

Myalgia { } Fever { } Fatigue { } Dry cough { }

1. **Sources of information about COVID-19**

Radio { } Television { } Social media { } Health workers { } Friend { } Religious leaders { } Magazines { } Newspapers { }

1. **Is your phone connected to the internet?**

Yes **{ }** No **{ }**

**Table 2: Knowledge about COVID-19**

| **S/N** | **Questions** | **Yes** | **No** | **I don’t know** |
| --- | --- | --- | --- | --- |
| 4 | Unlike the common cold, stuffy nose, runny nose, and sneezing are less common in persons infected with the COVID-19 virus |  |  |  |
| 5 | There is no effective cure for COVID-2019 at the moment. |  |  |  |
| 6 | Early symptomatic and supportive treatment can help most patients recover from the infection |  |  |  |
| 7 | Not all persons with COVID-2019 will develop to severe cases. |  |  |  |
| 8 | Elderly people, people with underlying chronic illnesses and obese are more likely to develop severe cases |  |  |  |
| 9 | Eating or contacting wild animals would result in the infection by the COVID-19 virus |  |  |  |
| 10 | Persons with COVID-2019 cannot infect the virus to others when a fever is not present |  |  |  |
| 11 | The COVID-19 virus spreads via respiratory droplets of infected individuals |  |  |  |
| 12 | Ordinary residents can wear general medical masks to prevent the infection by the COVID-19 virus |  |  |  |
| 13 | It is not necessary for children and young adults to take measures to prevent the infection by the COVID-19 virus |  |  |  |
| 14 | To prevent the infection by COVID-19, individuals should avoid going to crowded places |  |  |  |
| 15 | Isolation and treatment of people who are infected with the COVID-19 virus are effective ways to reduce the spread of the virus |  |  |  |
| 16 | People who have contact with someone infected with the COVID-19 virus should be immediately isolated in a proper place. In general, the observation period is 14 days |  |  |  |

**(Section B)**

The section contains questions on the attitude of the respondents toward COVID-19

**Instruction**: Tick appropriately

**Table 3: Attitude toward prevention of the spread COVID-19**

| **S/N** | **Questions** | **Yes** | **No** | **I don’t know** |
| --- | --- | --- | --- | --- |
| 1 | Do you agree that COVID-19 will finally be successfully controlled? |  |  |  |
| 2 | Do you have confidence that Uganda can win the battle against the COVID-19 virus? |  |  |  |
| 3 | I can confidently participate in the management of patients with COVID-19 |  |  |  |
| 4 | Hand washing can prevent you from contracting COVID-19 |  |  |  |
| 5 | Do you feel happy about the partial lock down by the government |  |  |  |

**(Section C)**

The section contains questions on the practices of the respondents toward COVID-19

**Instruction**: Tick appropriately

**Table 4: Practices toward prevention of the spread of COVID-19**

| **S/N** | **Questions** | **Yes** | **No** | **I don’t know** |
| --- | --- | --- | --- | --- |
| 1 | In recent days, have you gone to any crowded place, other than the market? |  |  |  |
| 2 | In recent days, have you worn a mask when leaving home? |  |  |  |
| 3 | Do you use hand sanitizer? |  |  |  |
| 4 | Do you wash your hands regularly? |  |  |  |
| 5 | Do you cover your mouth with handkerchief, elbow or tissue paper when sneezing? |  |  |  |
| 6 | Do you clean your floor and surfaces regularly with soap or bleach? |  |  |  |
| 7 | Do you observe social distance? |  |  |  |
